# 1381. Barriers and Facilitators to Prompt Diagnosis of Tuberculosis in Lima, Peru

**DOI:** 10.1093/ofid/ofad500.1218

**Published:** 2023-11-27

**Authors:** Carolina Geadas, Ana Karina Millones, Diana Acosta, Sheyla Farroñay, Isabel Torres, Judith Jimenez, Leonid Lecca, Courtney Yuen

**Affiliations:** Massachusetts General Hospital and Brigham and Women's Hospital, Boston, Massachusetts; Socios En Salud Sucursal Peru, Lima, Peru, Lima, Lima, Peru; Socios En Salud Sucursal Peru, Lima, Peru, Lima, Lima, Peru; Socios En Salud Sucursal Peru, Lima, Peru, Lima, Lima, Peru; Socios En Salud Sucursal Peru, Lima, Peru, Lima, Lima, Peru; Socios En Salud Sucursal Peru, Lima, Peru, Lima, Lima, Peru; Socios En Salud Sucursal Peru, Lima, Peru, Lima, Lima, Peru; Harvard Medical School

## Abstract

**Background:**

Tuberculosis (TB) infectiousness decreases significantly with only a few days of therapy, but delayed diagnosis often leads to late treatment initiation. We conducted a sequential explanatory mixed-methods study to understand the barriers and facilitators to prompt diagnosis and identify areas for improvement.

**Methods:**

We enrolled 100 adults who started TB therapy in the Carabayllo district of Lima, Peru, between November 2020 and February 2022 and administered a survey about their symptoms and healthcare encounters. We calculated the time from symptom onset to first healthcare visit and to diagnosis. We conducted semi-structured interviews of 26 participants who had a range of delays, investigating their experiences navigating the health system. Interview transcripts were inductively coded for concepts related to diagnostic barriers and facilitators and suggestions for improvement.

**Results:**

Overall, 38% of patients sought care from public facilities and 42% from the private sector. Only 33% reported being diagnosed with TB on their first visit. The median total diagnostic delay was 9 weeks (interquartile range [IQR] 4–22), with a median of 4 weeks (IQR 0–9) before contact with the health system and of 3 weeks (IQR 0–9) after. There was a median of 3 (IQR 2–4) health visits before diagnosis. Several factors contributed to a delayed diagnosis. Many patients attributed their symptoms to an alternative cause or had misconceptions about the disease, and often postponed seeking care. Once they sought care, the vast majority of patients had multiple encounters, sometimes alternating between the public and private sectors. Having a referral document and being seen by a pulmonologist often expedited the diagnosis. Several patients identified a need for more education about the disease through community campaigns and for better access to pulmonologists.

Factors impacting the time to diagnosis of tuberculosis in the Carabayllo district of Lima, Peru

**Figure:**
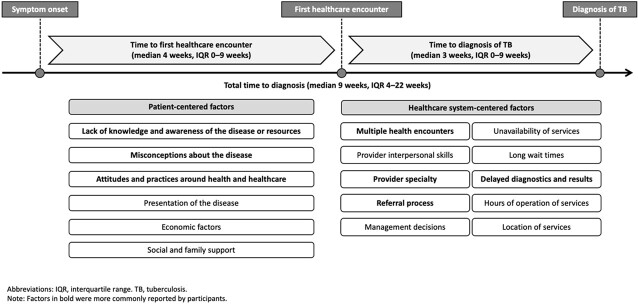

**Conclusion:**

We identified important diagnostic delays related to both patient- and health system-associated factors. Misinformation about TB, poor accessibility of health services, and the need for multiple encounters to obtain diagnostic tests were major factors leading to delays. Better strategies for community education and integration between the public and private sectors should be priorities in the efforts to combat TB.

**Disclosures:**

**All Authors**: No reported disclosures

